# Optimizing Outcomes: Bevacizumab with Carboplatin and Paclitaxel in 5110 Ovarian Cancer Patients—A Systematic Review and Meta-Analysis

**DOI:** 10.3390/ph17081095

**Published:** 2024-08-21

**Authors:** Yu Jin Kim, Hee Min Lee, Ga Eun Lee, Jin Hui Yoo, Hwa Jeong Lee, Sandy Jeong Rhie

**Affiliations:** 1 College of Pharmacy, Ewha Womans University, Seoul 03760, Republic of Korea; graceyujin@ewhain.net (Y.J.K.); gaeunlee333@ewha.ac.kr (G.E.L.); jhyoo1025@ewhain.net (J.H.Y.); hwalee@ewha.ac.kr (H.J.L.); 2 Graduate School of Life and Pharmaceutical Sciences, Ewha Womans University, Seoul 03760, Republic of Korea; hymina@ewhain.net; 3 Department of Pharmacy, Ewha Womans University Seoul Hospital, Seoul 07804, Republic of Korea; 4 Graduate School of Pharmaceutical Sciences, Ewha Womans University, Seoul 03760, Republic of Korea

**Keywords:** ovarian cancer, chemotherapy, carboplatin, paclitaxel, bevacizumab, efficacy, safety

## Abstract

Background/Objectives: The study aimed to evaluate the efficacy and safety of incorporating bevacizumab into the combination therapy of carboplatin and paclitaxel for epithelial ovarian cancer and other clinical applications. Methods: A systematic review was conducted following PRISMA guidelines using keyword searches in PubMed, Embase, Cochrane Library, CINAHL, ClinicalTrials.gov, and ICTRP until February 2024. Randomized controlled trials (RCTs) comparing carboplatin and paclitaxel with and without bevacizumab in ovarian cancer patients were included. Efficacy outcomes were overall survival (OS) and progression-free survival (PFS), as described by hazard ratios (HRs). Safety outcomes were analyzed with risk ratios (RRs) for 16 adverse events. Results: Seven RCTs (n = 5110) were included. The combination with bevacizumab significantly improved PFS (HR: 0.73; 95% confidence interval: 0.58, 0.92; *p* = 0.008). The chemotherapy group receiving bevacizumab with carboplatin and paclitaxel showed a significantly higher incidence of hypertension, non-CNS bleeding, thromboembolic events, GI perforation, pain, and proteinuria. Conclusions: The combination of carboplatin, paclitaxel, and bevacizumab improves PFS compared to the regimen without bevacizumab, but it raises significant safety concerns. Clinical management should consider adverse event prevention by vigilantly monitoring blood pressure, signs and symptoms of bleeding, thromboembolism, GI perforation, and pain to balance the therapeutic benefits with the potential risks of this combination therapy.

## 1. Introduction

Globally, ovarian cancer is the eighth most common cancer in women, accounting for 3.4% of cases and 4.8% of cancer deaths in 2022 [1]. Since the years recorded the highest in northern Europe and North America, the age-standardized incidence has declined in these regions and increased in parts of eastern Europe and Asia [2]. These trends are probably explained by the fact that more cancers have been coded as fallopian tube in origin, and also suggest that preventive surgical procedures and medicinal therapies are effective for the subject population in high-income countries [2].

In line with the previous review [2], cancer statistics in the U.S. show that ovarian cancer was the fifth leading cause of cancer death in 2021, and its incidence and mortality declined, concurrently with a 5-year relative survival rate of 51% in 2013–2019 [3]. Compared to Korea, ovarian cancer is rated as the eighth cause of annual cancer deaths in 2022, and its 5-year relative survival rate was 65.1% in 2016–2020, though the incidence rate has been increasing [4].

Over 95% of ovarian cancer is epithelial ovarian cancer (EOC), a heterogeneous disease encompassing five distinct histotypes. Among those, high-grade serous ovarian carcinoma is the most common histologic subtype, accounting for over 70% of EOCs [5,6]. Currently, high-grade serous ovarian carcinomas are recognized to originate from the fallopian tube [5,7], making changes in coding, reporting, and incidence trends difficult to interpret over the past decade [2]. Moreover, although early-stage disease is highly curable [5,8], the disease presents mainly in stages III/IV. Additionally, mortality due to late-stage ovarian cancer is over 75% [5,9,10].

The combination therapy of carboplatin and paclitaxel, often further combined with bevacizumab, is one of the primary first-line treatments for ovarian cancer [11]. In June 2018, the Food and Drug Administration (FDA) in the United States (U.S.) approved bevacizumab in combination with carboplatin and paclitaxel, followed by single-agent bevacizumab, for stage III or IV ovarian carcinoma patients after initial surgical resection [12,13]. With the results of phase III trials such as GOC-0218 and ICON7, which drove approval for more indications [12], the NCCN guideline recommends paclitaxel/carboplatin/bevacizumab + maintenance bevacizumab therapy as the preferred regimen in primary care for stage II–IV disease [11]. Based on this guidance, the most common frontline chemotherapy backbone and route of administration for systemic treatment remains intravenous carboplatin plus paclitaxel. To date, combining targeted therapy with antiangiogenics is a promising strategy [11]. However, the best candidate population for adjuvant bevacizumab treatment prescription is still a subject of controversy [12], since no benefit in overall survival (OS) with bevacizumab was found in the final survival analysis report of GOG-0218 and an additional analysis of the ICON7 trial reported by Oza et al., demonstrating the benefit in OS with bevacizumab in the specific predefined high-risk population.

With this limited benefit of overall survival, there are certain safety concerns, although bevacizumab is included in the standard chemotherapy and is anticipated to inhibit cancer recurrence and neovascularization necessary for tumor nourishment. While previous studies reported the safety profile of the combination therapy of carboplatin, paclitaxel, and bevacizumab, a comprehensive analysis of adverse events remains insufficient to apply this treatment in clinical settings [14,15,16,17,18,19].

These epidemiologic and prognostic circumstances are not convincing for healthcare providers and patients to make decisions about treatment options such as bevacizumab incorporation into standard chemotherapy. It necessitates comprehensive analyses in which the study population encompasses a broad range of cancer stages and histologic types regardless of whether they are relapsed or newly diagnosed, and almost all the adverse events reported on published data are collected without loss of possible toxicities. Thus, our study aims to evaluate both the efficacy and safety of the treatment through meta-analysis, with the selection of the latest and larger cohort studies eligible for selection with pre-specified criteria that require targeted therapy of bevacizumab as an intervention added to standard chemotherapy of carboplatin and paclitaxel.

## 2. Materials and Methods

### 2.1. Data Sources and Search Strategy

This systematic review was submitted and registered in the International Prospective Register of Systematic Reviews (PROSPERO) database (CRD42024538443). This study was conducted according to the Preferred Reporting Items for Systematic Reviews and Meta-Analyses (PRISMA) guidelines [20]. We searched MEDLINE (PubMed), Embase, the Cochrane Central Register of Controlled Trials (CENTRAL), the Cumulative Index to Nursing and Allied Health Literature (CINAHL), ClinicalTrials.gov, and the International Clinical Trials Registry Platform (ICTRP) without year and language limitations, focusing on randomized controlled trials (RCTs). We used a combination of keywords and medical subject headings (MeSHs): ‘bevacizumab’, ‘carboplatin’, ‘paclitaxel’, and ‘ovarian cancer’. The last search was conducted in February 2024. Additionally, we manually searched the references of eligible articles to identify additional studies for meta-analysis.

### 2.2. Study Selection and Data Extraction

Two independent reviewers screened the titles and abstracts of all studies identified in the database search to verify their eligibility. Disagreements about the study selection were resolved through discussion with a third reviewer if a consensus was not reached. Duplicated studies and clinical trial protocols were excluded.

We extracted study characteristics (author, publication year, study duration, study region, study design, and clinical study phase), study population (ovarian cancer stage and number of patients for each treatment arm), study interventions and comparators (medication names, dosage, and schedule), and efficacy and safety outcomes from eligible studies. Efficacy outcomes included overall survival (OS) and progression-free survival (PFS). Safety outcomes included adverse events such as hypertension, heart failure, central nervous system (CNS) bleeding, non-CNS bleeding, thromboembolic events (any, arterial, or venous), neutropenia, febrile neutropenia, anemia, wound complications, gastrointestinal (GI) disorders, GI perforation, pain, dermatologic disorders, and proteinuria. Table 1 describes the PICOS (patients, intervention, comparator, outcomes, and study design) summary of our study.

### 2.3. Assessment of Bias Risk and Evidence

Two independent reviewers assessed the methodological quality of RCTs using the Cochrane’s Risk of Bias (RoB) tool [21]. Studies were evaluated as low, unclear, or high risk in the following domains: random sequence generation, allocation concealment, blinding of participants and personnel, blinding of outcome assessment, incomplete outcome data, selective reporting, and other biases. Disagreements on the risk of bias and the quality of evidence were resolved by consensus or a third reviewer if consensus was not met (Supplement Figure S1).

### 2.4. Statistical Analysis

OS and PFS were analyzed with a hazard ratio (HR) and a 95% confidence interval (CI). The risks of adverse events for safety evaluation were analyzed by relative risks (RRs) and 95% CIs. Heterogeneity across the included studies was evaluated by Cochran’s Q test (*p* < 0.10 was considered significant) [22] and the quantified I^2^ index [23]. Outcomes with high heterogeneity (I^2^ > 50%) were analyzed by a random-effect model of the inverse variance (IV) method. Those with low heterogeneity (I^2^ < 50%) were analyzed by a fixed-effect model. *p*-values < 0.05 indicated statistical significance. All statistical analyses were conducted using Review Manager (RevMan) Version 5.4.1 (The Cochrane Collaboration, 2020, London, UK).

## 3. Results

### 3.1. Study Search and Selection

**Figure 1:**
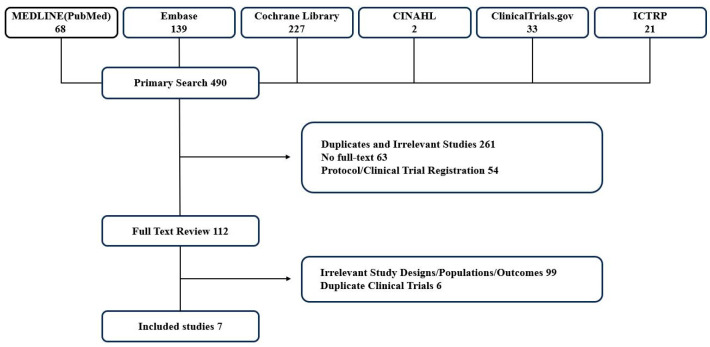
Study selection diagram (Preferred Reporting Items for Systematic Reviews and Meta-Analyses, PRISMA).

The primary literature search yielded 490 studies (Figure 1). A total of 112 studies were included for full-text review after excluding duplicates or irrelevant studies (n = 261), studies without available full-text (n = 63), and study protocols or clinical trial registrations (n = 54). After further exclusion based on irrelevant study designs, populations, and outcomes (n = 99), as well as duplicate clinical trials (n = 6), seven studies were eligible for analysis.

Thus, these seven RCTs were selected for our meta-analysis based on the inclusion criteria according to the definition of PICOS.

### 3.2. Study Characteristics

The baseline characteristics of the study populations, such as age and Eastern Cooperative Oncology Group (ECOG) status, were well balanced between the arms in the original studies. The study regions included the United States, Canada, Japan, South Korea, the United Kingdom, Australia, France, Germany, Norway, New Zealand, Denmark, Finland, Sweden, Spain, Greece, Italy, Monaco, and Switzerland. The number of ovarian cancer patients used in our meta-analysis was 5110, satisfying the needs of the meta-analysis. Five RCTs were used for efficacy analysis, and five RCTs were used for safety analysis (Table 2).

In the extracted studies, this systematic review attempted to analyze as many areas of adverse events as possible, if comparable. While combining the statistical values of RCTs, ‘names with the same scope’ were grouped into categories to conduct an accurate meta-analysis. For example, thromboembolic events were subdivided into ‘Any Thromboembolic Events’, ‘Arterial Thromboembolic Events’, and ‘Venous Thromboembolic Events’ and analyzed using only statistical values exactly corresponding to the scope of the name. The reason for the analysis by dividing it into ‘Neutropenia’ and ‘Febrile Neutropenia’ was the same. Furthermore, for example, constipation and diarrhea are relatively detailed names, but they were excluded because there was no comparison target. Instead, the statistical value specified as ‘GI disorders’ had enough RCT studies to proceed with the comparison. The corresponding meta-analysis was conducted using statistical values of ‘GI events’, ‘Gastrointestinal’, and ‘Gastrointestinal disorders’, which correspond to the exact size of the scope of the name.

Oza 2015 and Perren 2011 were the same clinical trials (ICON7) used in this paper. Oza 2015, which was more recent, was used for the efficacy analysis. For the safety analysis, Perren 2011 provided results for more adverse event categories than Oza 2015, with specific and clear numbers including the exact number of total patients; hence, Perren 2011 was used instead of Oza 2015.

### 3.3. Efficacy Outcomes

**Figure 2:**
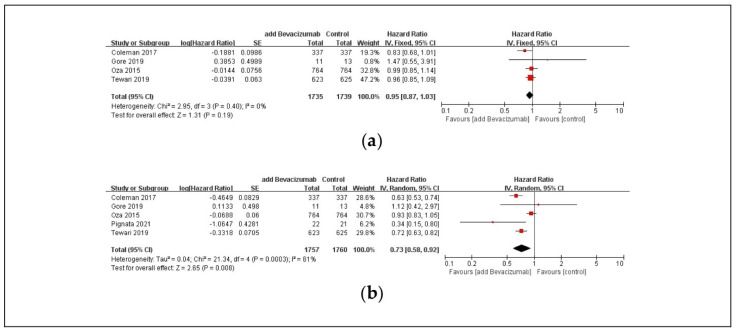
Forest plot of (**a**) OS and (**b**) PFS in ovarian cancer patients. Abbreviations: CI, confidence interval; IV, inverse variance; OS, overall survival; PFS, progression-free survival [24,25,26,28,30].

OS did not differ significantly between the two treatment groups (HR: 0.95; 95% Cl: 0.87, 1.03; *p* = 0.19, Figure 2a). However, the incidence of events (disease progression or death) was 0.73 times in the combination therapy including bevacizumab compared to the carboplatin and paclitaxel control group (HR: 0.73; 95% Cl: 0.58, 0.92; *p* = 0.008, Figure 2b).

### 3.4. Safety Outcomes

**Figure 3:**
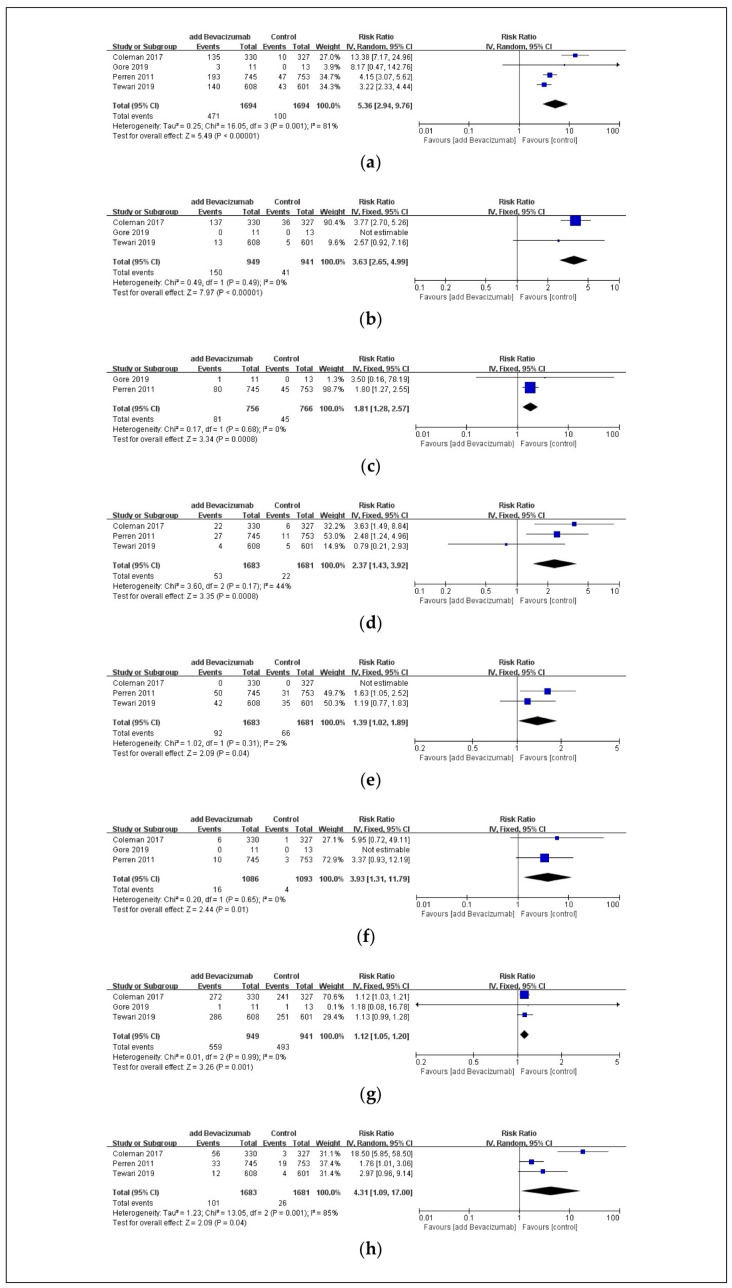
Forest plot of adverse events associated with chemotherapy in ovarian cancer patients. These adverse events are (**a**) hypertension, (**b**) non-CNS bleeding, (**c**) any thromboembolic events, (**d**) arterial thromboembolic events, (**e**) venous thromboembolic events, (**f**) GI perforation, (**g**) pain, and (**h**) proteinuria. Abbreviations: CI, confidence interval; CNS, central nervous system; GI, gastrointestinal; IV, inverse variance [24,25,27,30].

The combination therapy, including bevacizumab, was associated with a significantly higher incidence of the following safety outcomes compared to the carboplatin and paclitaxel control groups: hypertension (RR: 5.36; 95% CI: 2.94, 9.76; *p* < 0.00001, Figure 3a), non-CNS bleeding (RR: 3.63; 95% CI: 2.65, 4.99; *p* < 0.00001, Figure 3b), any thromboembolic events (RR: 1.81; 95% CI: 1.28, 2.57; *p* = 0.0008, Figure 3c), arterial thromboembolic events (RR: 2.37; 95% CI: 1.43, 3.92; *p* = 0.0008, Figure 3d), venous thromboembolic events (RR: 1.39; 95% CI: 1.02, 1.89; *p* = 0.04, Figure 3e), GI perforation (RR: 3.93; 95% CI: 1.31, 11.79; *p* = 0.01, Figure 3f), pain (RR: 1.12; 95% CI: 1.05, 1.20; *p* = 0.001, Figure 3g), and proteinuria (RR: 4.31; 95% CI: 1.09, 17.00; *p* = 0.04, Figure 3h).

**Figure 4:**
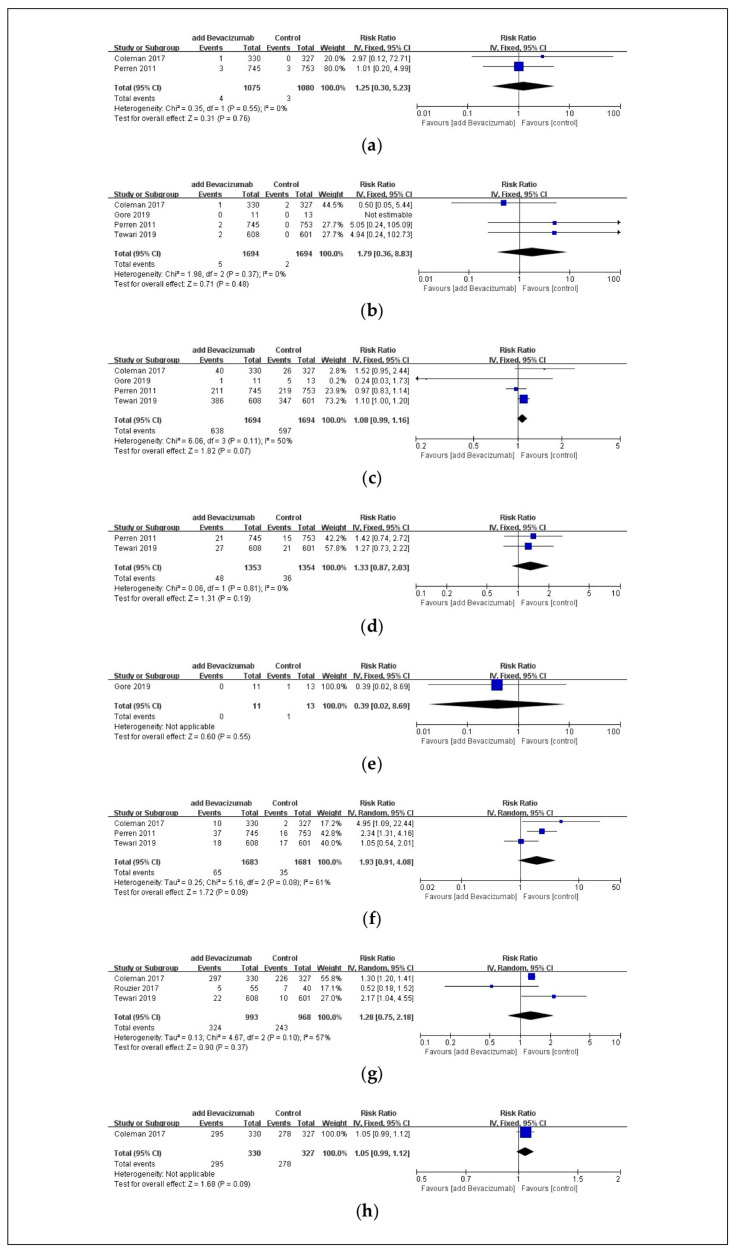
Forest plot of adverse events without significant difference in ovarian cancer patients. These adverse events are (**a**) heart failure, (**b**) CNS bleeding, (**c**) neutropenia, (**d**) febrile neutropenia, (**e**) anemia, (**f**) wound complications, (**g**) GI disorders, and (**h**) dermatologic disorders. Abbreviations: CI, confidence interval; CNS, central nervous system; GI, gastrointestinal; IV, inverse variance [24,25,27,29,30].

There was no significant difference in the incidence of heart failure (Figure 4a), CNS bleeding (Figure 4b), neutropenia (Figure 4c), febrile neutropenia (Figure 4d), anemia (Figure 4e), wound complications (Figure 4f), GI disorders (Figure 4g), and dermatologic disorders (Figure 4h) between the two groups.

## 4. Discussion

The efficacy outcome in this study demonstrated that bevacizumab (15 or 7.5 mg/kg) incorporation into standard chemotherapy (carboplatin (AUC 5 or 6) and paclitaxel (175 mg/m^2^)) improved progression-free survival, but the overall survival benefit is not statistically significant compared to the doublet of carboplatin and paclitaxel alone. This result is the same as those from previous studies that drove the regulatory authorities’ approval of bevacizumab for most indications and frontline use for later stages of ovarian cancer without proof of OS benefit, but only with improved PFS [24,30].

In previous studies for efficacy evaluation, OS was mostly insignificant [14,15,16], but in some studies [17,31], there were significant results. PFS improved significantly or was not significant, depending on the situation [15]. Regarding the reasons that OS benefit has been hardly proven, Coleman (2017) noted the morphologies of OS curves in two groups (i.e., chemotherapy alone vs. chemotherapy plus bevacizumab). The curves since randomization closely approximate each other until 22 months, and then diverge with increasing death events [24]. The investigator proposed that the specific patterns might be related to relatively few deaths in early treatment, drug–drug interaction between paclitaxel and bevacizumab, and the efficacy of maintenance bevacizumab [24,32]. Pfisterer (2023) conducted a clinical trial to extend the duration of bevacizumab for PFS and OS (primary and secondary endpoints, respectively) and concluded that longer treatment with bevacizumab for up to 30 months did not improve PFS or OS in patients with primary epithelial ovarian, fallopian tube, or peritoneal cancer. The bevacizumab treatment duration of 15 months remains the standard of care [33]. In the FDA ovarian cancer clinical trial endpoint workshop, a consensus opinion was that surrogate endpoints for accelerated approval of novel agents in certain circumstances should precede the approval of the front-line use of bevacizumab with study GOG-0218 results [34].

Regardless of these disease and drug-specific issues, PFS has been an attractive endpoint because, compared with OS, studies may be conducted more quickly using fewer subjects and at lower costs, but the direct measurement of both PFS and quality of life (QOL) may be a practical and informative alternative when measurement of OS is unfeasible [35]. On the other hand, patients’ QOL cannot be assumed from PFS [36]. It is not known whether patients would exactly understand what PFS actually means without an explanation that PFS may not predict OS [35]. At that point, our study is meaningful to evaluate both safety and efficacy, weighing the adverse event analysis. The risk rates of hypertension, non-CNS bleeding, thromboembolic events, GI perforation, pain, and proteinuria were higher with bevacizumab-containing combination therapy compared to therapies without bevacizumab. Even though these are consistent with the adverse events known as monoclonal antibody infusion reaction and target-related side effects of bevacizumab, it should not be neglected to consider that adverse events such as pain, thromboembolism, and cardio- and nephrotoxicity, which are also associated with carboplatin or paclitaxel, could be more severe or occur more frequently with the concomitant use of bevacizumab. Therefore, stringent safety considerations should be warranted for patients with pre-existing cardiovascular and GI diseases to ensure optimal treatment outcomes in the clinical setting. Identifying the characteristics of each patient, such as underlying diseases, allergies, and concomitant drugs, is necessary. The medical team should monitor patients carefully to prevent adverse events and take appropriate measures according to the individual’s condition and characteristics when the adverse event occurs. Complying with guidelines, initiating bevacizumab-containing combination therapy should be delayed for at least 28 days after significant surgery or until complete healing of surgical sites to avoid bleeding [13]. Additionally, the therapy should be discontinued in cases of GI perforation, tracheoesophageal fistula, or any grade 4 fistula [13]. Regular urine tests are necessary for proteinuria monitoring [13]. Moreover, ambulatory or home blood pressure monitoring is recommended, with therapy cessation advised for systolic blood pressure ≧160 mmHg or diastolic blood pressure ≧100 mmHg [37]. Angiotensin-converting enzyme inhibitors are recommended as the first-line treatment to prevent hypertension-related proteinuria due to the combination chemotherapy containing bevacizumab, which might increase the release of nitric oxide, dilating blood vessels [38]. Dihydropyridine calcium channel blockers are effective in managing refractory hypertension by reducing the contraction of vascular smooth muscle cells in hypercontractile blood vessels caused by nitric oxide signaling impairment induced by vascular endothelial factor inhibitors [39].

The limitations of this study include small data on heart failure, thromboembolic events, febrile neutropenia, anemia, and dermatologic events due to an inadequate number of clinical trials eligible for this study restricted to intervention with a combination of carboplatin, paclitaxel, and bevacizumab. Moreover, one of the selected studies was translated by Coleman (2017) for clinical trial GOG-0213, which was designed for recurrent OC subjects with completion of front-line chemotherapy (both platinum and taxane); front-line therapy may have included a biologic agent (i.e., bevacizumab). The exclusion criteria in GOG-0213 were set up for conditions that might be adverse events from previous systemic therapy, such as bowel obstruction and perforation, peripheral neuropathy and CNS disease, bleeding disorder, coagulopathy, an allergic reaction to carboplatin and/or paclitaxel, bevacizumab, and cardiovascular disease. Consequently, we could infer that the statistically significant adverse events analyzed, including the study by Coleman (2017), have much greater risk potential, although other adverse events demonstrated by this analysis as not significant could not be ignored. Heart failure is a potentially fatal disease, emphasizing the critical nature of its management during chemotherapy. Concurrently, wound complications can significantly delay postsurgical recovery. Furthermore, anemia and dermatologic disorders can profoundly affect the patient’s quality of life throughout chemotherapy.

Despite limitations, unlike other systematic reviews [14,15,16,17,18,19,31], we provided a detailed classification of large and small categories (e.g., GI disorders and GI perforation, respectively) to investigate adverse events comprehensively and in detail. This avoids missing specific adverse event profiles (for example, GI perforation was associated with bevacizumab-containing therapy), guiding focused monitoring and management strategies. Moreover, we analyzed 16 adverse events, which is more than in previous studies [14,15,16,17,18,19,31]. Furthermore, this study significantly contributes to the clinical understanding of related safety concerns. We also analyzed various previously underexplored adverse events, including heart failure, anemia, wound complications, and dermatologic events. We performed a comprehensive meta-analysis using risk ratios and forest plots that methodologically helped compare the risks of the two groups. Additionally, in this study, we raised the level of evidence compared to prior research by including larger patient cohorts [14,15,16,17,18,19,31]. In contrast to the latest systematic review paper that analyzed some of the areas covered in this study and conducted the search until September 2022 [15], the relevance of this study is emphasized by its incorporation of the latest data search until February 2024.

Our study team expected that this review would help respective patients take systemic therapy, ensuring safety when utilizing personalized medication. Moreover, this would help healthcare providers make decisions about what they aim for and focus on during treatment by clarifying the limitations of the expected benefit of efficacy outcomes and weighing the active monitoring of significant adverse events of the combination therapy of carboplatin, paclitaxel, and bevacizumab.

## 5. Conclusions

The progression-free survival was significantly improved by adding bevacizumab to the combination therapy of carboplatin and paclitaxel. Nonetheless, OS did not show a significant difference. The risks of adverse events including hypertension, non-CNS bleeding, thromboembolic events, GI perforation, pain, and proteinuria increased significantly higher in the case of bevacizumab added to therapy than carboplatin and paclitaxel doublet alone. Therefore, careful monitoring and supportive care should be facilitated to avoid underestimation and inadequate treatment of potential overlapping adverse events in concomitant use of cytotoxic and antiangiogenic agents as part of efforts to maintain the quality of life in patients with ovarian cancer.

## Figures and Tables

**Table 1:** PICOS of this study.

Component	Definition
P (patients)	Patients with ovarian cancer
I (intervention)	Combination therapy of carboplatin, paclitaxel, and bevacizumab
C (comparator)	Combination therapy of carboplatin and paclitaxel
O (outcomes)	Efficacy: OS and PFS; safety (adverse events): hypertension, heart failure, CNS bleeding, non-CNS bleeding, thromboembolic events (any, arterial, or venous), neutropenia, febrile neutropenia, anemia, wound complications, GI disorders, GI perforation, pain, dermatologic disorders, and proteinuria
S (study design)	RCTs

Abbreviations: CNS, central nervous system; GI, gastrointestinal; OS, overall survival; PFS, progression-free survival; RCT, randomized clinical trial.

**Table 2:** Characteristics of the included studies.

Study Name	Study Period	Country	Study Design	Patient Population	Intervention	Comparator	OS Outcome	PFS Outcome	Safety Outcomes
Coleman 2017 [24]/GOG-0213 study	December 2007–November 2014	United States, Japan, and South Korea	Multi-center, open-label, randomized phase 3 trial	Adult females (aged ≥ 18 years) with recurrent measurable or evaluable epithelial ovarian, primary peritoneal, or fallopian tube cancer (n = 674)	Standard chemotherapy regimen plus bevacizumab (15 mg/kg) every 3 weeks, which was then continued as maintenance every 3 weeks until disease progression or unacceptable toxicity	Standard chemotherapy (six 3-weekly cycles of paclitaxel [175 mg/m^2^] and carboplatin [AUC 5])	HR: 0.829 [0.683–1.005], *p* = 0.056	HR: 0.628 [0.534–0.739], *p* < 0.0001	Hypertension, heart failure, CNS bleeding, non-CNS bleeding, arterial thromboembolic events, venous thromboembolic events, neutropenia, wound complications, GI disorders, GI perforation, pain, dermatologic disorders, and proteinuria
Gore 2019 [25]/GOG-0241 study	March 2010–August 2013	United Kingdom and the United States	Multi-center phase 3 factorial trial	Patients with histological diagnosis of primary mEOC; aged ≥ 18 years; newly diagnosed FIGO stage II–IV, or recurrence after stage I disease; no previous chemotherapy; ECOG performance status 0–2 (n = 24)	Carboplatin, paclitaxel, and bevacizumab (15 mg/kg intravenous every 3 weeks); then, bevacizumab maintenance (15 mg/kg on day 1, every 3 weeks)	Carboplatin (AUC 5/6) and paclitaxel (175 mg/m^2^), both intravenous, day 1	HR: 1.47, *p* = 0.44	HR: 1.12, *p* = 0.82	Hypertension, CNS bleeding, non-CNS bleeding, any thromboembolic events, neutropenia, anemia, GI perforation, and pain
Oza 2015 [26]/ICON7 study	April 2006–March 2013	11 countries across Europe, Canada, Australia, and New Zealand	International, phase 3, open-label, randomized trial	Patients aged ≥ 18 years; with newly diagnosed epithelial ovarian, fallopian tube, or primary peritoneal cancer; an ECOG performance status of 0–2; FIGO stage IIb–IV or high-risk (grade 3 or clear cell histology) stage I–IIa disease (n = 1528)	Standard chemotherapy plus intravenous bevacizumab 7.5 mg/kg every 3 weeks given concurrently and continued with up to 12 further 3-weekly cycles of maintenance therapy	Standard chemotherapy (six 3-weekly cycles of intravenous carboplatin [AUC 5 or 6] and paclitaxel 175 mg/m^2^)	HR: 0.99 [0.85–1.14], *p* = 0.85	HR: 0.93 [0.83–1.05], *p* = 0.25	Not available
Perren 2011 [27]/ICON7 study	April 2006–February 2010	United Kingdom, Germany, France, Canada, Australia, New Zealand, Denmark, Finland, Norway, Sweden, and Spain	Phase 3, open-label, randomized trial	Females with histologically confirmed, high-risk, early-stage disease (FIGO stage I or IIA and clear-cell or grade 3 tumors) or advanced (FIGO stage IIB to IV) epithelial ovarian cancer, primary peritoneal cancer, or fallopian tube cancer (n = 1498)	Standard-chemotherapy group plus bevacizumab (7.5 mg/kg) given concurrently every 3 weeks for 5 or 6 cycles and continued for 12 additional cycles or until disease progression	Carboplatin (AUC 5 or 6) and paclitaxel (175 mg/m^2^) given every 3 weeks for 6 cycles (standard-chemotherapy group)	Not available	Not available	Hypertension, heart failure, CNS bleeding, any thromboembolic events, arterial thromboembolic events, venous thromboembolic events, neutropenia, febrile neutropenia, wound complications, GI perforation, and proteinuria
Pignata 2021 [28]/NCT01802749 study	December 2013–February 2018	France, Greece, Italy, Monaco, and Switzerland	Academic, multi-center, open-label, randomized phase 3 trial	Females aged ≥ 18 years with histologically confirmed FIGO stage IIIB–IV ovarian cancer, fallopian tube carcinoma, or peritoneal carcinoma (including mixed Mullerian tumors) (n = 43)	Carboplatin-based doublet plus bevacizumab	Intravenous carboplatin-based doublet (carboplatin AUC 5 on day 1 plus paclitaxel 175 mg/m^2^ on day 1, every 21 days	Not available	HR: 0.34 [0.15–0.80]	Not available
Rouizer 2017 [29]/ANTHALYA study	January 2013–August 2016	France	Multi-center, open-label, non-comparative phase 2 study	Females aged ≥ 18 years with histologically confirmed, chemotherapy-naive, high-risk FIGO stage IIIC/IV epithelial ovarian carcinoma, fallopian tube carcinoma, or primary peritoneal carcinoma (ineligible for primary complete debulking surgery) (n = 95)	Four cycles of neoadjuvant carboplatin-paclitaxel + 3 concomitant cycles of bevacizumab 15 mg/kg	Four cycles of neoadjuvant carboplatin-paclitaxel	Not available	Not available	GI disorders
Tewari 2019 [30]/GOG-0218 study	October 2005–January 2018	United States, Canada, Japan, and South Korea	International, multi-center, double-blind, placebo-controlled, phase 3 trial	Females with stage III or IV epithelial ovarian, primary peritoneal, or fallopian tube carcinoma (n = 1248)	Six 21-day cycles of intravenous carboplatin (AUC 6) plus paclitaxel (175 mg/m^2^), followed by bevacizumab (15 mg/kg) in cycles 7 to 22	Six 21-day cycles of intravenous carboplatin (AUC 6) plus paclitaxel (175 mg/m^2^), followed by 16 21-day cycles of placebo	HR: 0.96 [0.85–1.09], *p* = 0.53	HR: 0.717 [0.625–0.824], *p* < 0.001	Hypertension, CNS bleeding, non-CNS bleeding, arterial thromboembolic events, venous thromboembolic events, neutropenia, febrile neutropenia, wound complications, GI disorders, pain, and proteinuria

Abbreviations: AUC, area under the curve; CNS, central nervous system; ECOG, Eastern Cooperative Oncology Group; FIGO, International Federation of Gynecology and Obstetrics; GI, gastrointestinal; HR, hazard ratio; mEOC, mucinous epithelial ovarian cancer. Note: The OS and PFS Outcome columns represent the HR [95% CI] and *p* value.

## Data Availability

Data derived from public domain resources.

## Supplementary Materials

The following supporting information can be downloaded at: https://www.mdpi.com/article/10.3390/ph17081095/s1: Figure S1: Risk of bias in the included studies.

## References

[1] Bray F., Laversanne M., Sung H., Ferlay J., Siegel R.L., Soerjomataram I., Jemal A. (2024). Global cancer statistics 2022: GLOBOCAN estimates of incidence and mortality worldwide for 36 cancers in 185 countries. CA A Cancer J. Clin..

[2] Webb P.M., Jordan S.J. (2024). Global epidemiology of epithelial ovarian cancer. Nat. Rev. Clin. Oncol..

[3] Siegel R.L., Giaquinto A.N., Jemal A. (2024). Cancer statistics, 2024. CA A Cancer J. Clin..

[4] KOSIS (2021). Relative Survival Rate for 5 Years by 24 Kinds of Cancer, Cancer Occurrence Time and Gender. https://kosis.kr/statHtml/statHtml.do?orgId=117&tblId=DT_117N_A00021&conn_path=I2&language=en.

[5] Lheureux S., Braunstein M., Oza A.M. (2019). Epithelial ovarian cancer: Evolution of management in the era of precision medicine. CA A Cancer J. Clin..

[6] Prat J. (2012). Ovarian carcinomas: Five distinct diseases with different origins, genetic alterations, and clinicopathological features. Virchows Arch..

[7] Kurman R. (2013). Origin and molecular pathogenesis of ovarian high-grade serous carcinoma. Ann. Oncol..

[8] Peres L.C., Cushing-Haugen K.L., Köbel M., Harris H.R., Berchuck A., Rossing M.A., Schildkraut J.M., Doherty J.A. (2019). Invasive epithelial ovarian cancer survival by histotype and disease stage. JNCI J. Natl. Cancer Inst..

[9] Banerjee S., Kaye S.B. (2013). New strategies in the treatment of ovarian cancer: Current clinical perspectives and future potential. Clin. Cancer Res..

[10] Ghirardi V., Fagotti A., Ansaloni L., Valle M., Roviello F., Sorrentino L., Accarpio F., Baiocchi G., Piccini L., De Simone M. (2023). Diagnostic and therapeutic pathway of advanced ovarian cancer with peritoneal metastases. Cancers.

[11] NCCN (2023). Ovarian Cancer. NCCN Guidelines for Patients.

[12] Colomban O., Tod M., Peron J., Perren T.J., Leary A., Cook A.D., Sajous C., Freyer G., You B. (2020). Bevacizumab for newly diagnosed ovarian cancers: Best candidates among high-risk disease patients (ICON-7). JNCI Cancer Spectr..

[13] (2022). Genentech. Avastin. Highlights of Prescribing Information. https://www.accessdata.fda.gov/drugsatfda_docs/label/2022/125085s340lbl.pdf.

[14] Aravantinos G., Pectasides D. (2014). Bevacizumab in combination with chemotherapy for the treatment of advanced ovarian cancer: A systematic review. J. Ovarian Res..

[15] Gaitskell K., Rogozińska E., Platt S., Chen Y., Abd El Aziz M., Tattersall A., Morrison J. (2023). Angiogenesis inhibitors for the treatment of epithelial ovarian cancer. Cochrane Database Syst. Rev..

[16] Hirte H., Poon R., Yao X., May T., Ethier J.-L., Petz L., Speakman J., Elit L. (2021). Neoadjuvant and adjuvant systemic therapy for newly diagnosed stage II-IV epithelial ovary, fallopian tube, or primary peritoneal carcinoma: A systematic review. Crit. Rev. Oncol./Hematol..

[17] Liu Y., Huang Y., Li J., Wan S., Jiang N., Yang J., Chiampanichayakul S., Tima S., Anuchapreeda S., Wu J. (2022). A comprehensive comparison of medication strategies for platinum-sensitive recurrent ovarian cancer: A Bayesian network meta-analysis. Front. Pharmacol..

[18] Petrillo M., Nero C., Carbone V., Bruno M., Scambia G., Fagotti A. (2018). Systematic review of cytoreductive surgery and bevacizumab-containing chemotherapy in advanced ovarian cancer: Focus on safety. Ann. Surg. Oncol..

[19] Song L., Liu Y., Chen Z., Li Z., Zhu S., Zhao Y., Li H. (2023). Association of bevacizumab and stroke in ovarian cancer: A systematic review and meta-analysis. Front. Neurosci..

[20] Moher D., Liberati A., Tetzlaff J., Altman D.G., PRISMA Group (2009). Preferred reporting items for systematic reviews and meta-analyses: The PRISMA statement. Ann. Intern. Med..

[21] Higgins J.P., Altman D.G., Gøtzsche P.C., Jüni P., Moher D., Oxman A.D., Savović J., Schulz K.F., Weeks L., Sterne J.A. (2011). The Cochrane Collaboration’s tool for assessing risk of bias in randomised trials. BMJ.

[22] Cochran W.G. (1954). The combination of estimates from different experiments. Biometrics.

[23] Higgins J.P., Thompson S.G., Deeks J.J., Altman D.G. (2003). Measuring inconsistency in meta-analyses. BMJ.

[24] Coleman R.L., Brady M.F., Herzog T.J., Sabbatini P., Armstrong D.K., Walker J.L., Kim B.G., Fujiwara K., Tewari K.S., O′Malley D.M. (2017). Bevacizumab and paclitaxel-carboplatin chemotherapy and secondary cytoreduction in recurrent, platinum-sensitive ovarian cancer (NRG Oncology/Gynecologic Oncology Group study GOG-0213): A multicentre, open-label, randomised, phase 3 trial. Lancet Oncol..

[25] Gore M., Hackshaw A., Brady W.E., Penson R.T., Zaino R., McCluggage W.G., Ganesan R., Wilkinson N., Perren T., Montes A. (2019). An international, phase III randomized trial in patients with mucinous epithelial ovarian cancer (mEOC/GOG 0241) with long-term follow-up: And experience of conducting a clinical trial in a rare gynecological tumor. Gynecol. Oncol..

[26] Oza A.M., Cook A.D., Pfisterer J., Embleton A., Ledermann J.A., Pujade-Lauraine E., Kristensen G., Carey M.S., Beale P., Cervantes A. (2015). Standard chemotherapy with or without bevacizumab for women with newly diagnosed ovarian cancer (ICON7): Overall survival results of a phase 3 randomised trial. Lancet Oncol..

[27] Perren T.J., Swart A.M., Pfisterer J., Ledermann J.A., Pujade-Lauraine E., Kristensen G., Carey M.S., Beale P., Cervantes A., Kurzeder C. (2011). A phase 3 trial of bevacizumab in ovarian cancer. N. Engl. J. Med..

[28] Pignata S., Lorusso D., Joly F., Gallo C., Colombo N., Sessa C., Bamias A., Salutari V., Selle F., Frezzini S. (2021). Carboplatin-based doublet plus bevacizumab beyond progression versus carboplatin-based doublet alone in patients with platinum-sensitive ovarian cancer: A randomised, phase 3 trial. Lancet Oncol..

[29] Rouzier R., Gouy S., Selle F., Lambaudie E., Floquet A., Fourchotte V., Pomel C., Colombo P.E., Kalbacher E., Martin-Francoise S. (2017). Efficacy and safety of bevacizumab-containing neoadjuvant therapy followed by interval debulking surgery in advanced ovarian cancer: Results from the ANTHALYA trial. Eur. J. Cancer.

[30] Tewari K.S., Burger R.A., Enserro D., Norquist B.M., Swisher E.M., Brady M.F., Bookman M.A., Fleming G.F., Huang H., Homesley H.D. (2019). Final Overall Survival of a Randomized Trial of Bevacizumab for Primary Treatment of Ovarian Cancer. J. Clin. Oncol..

[31] Ruan G., Ye L., Liu G., An J., Sehouli J., Sun P. (2018). The role of bevacizumab in targeted vascular endothelial growth factor therapy for epithelial ovarian cancer: An updated systematic review and meta-analysis. OncoTargets Ther..

[32] Poveda A.M., Selle F., Hilpert F., Reuss A., Savarese A., Vergote I., Witteveen P., Bamias A., Scotto N., Mitchell L. (2015). Bevacizumab combined with weekly paclitaxel, pegylated liposomal doxorubicin, or topotecan in platinum-resistant recurrent ovarian cancer: Analysis by chemotherapy cohort of the randomized phase III AURELIA trial. J. Clin. Oncol..

[33] Pfisterer J., Joly F., Kristensen G., Rau J., Mahner S., Pautier P., El-Balat A., Kurtz J.-E., Canzler U., Sehouli J. (2023). Optimal treatment duration of bevacizumab as front-line therapy for advanced ovarian cancer: AGO-OVAR 17 BOOST/GINECO OV118/ENGOT Ov-15 open-label randomized phase III trial. J. Clin. Oncol..

[34] Herzog T.J., Ison G., Alvarez R.D., Balasubramaniam S., Armstrong D.K., Beaver J.A., Ellis A., Tang S., Ford P., McKee A. (2017). FDA ovarian cancer clinical trial endpoints workshop: A Society of Gynecologic Oncology white paper. Gynecol. Oncol..

[35] Gutman S., Piper M., Grant M., Basch E., Oliansky D., Aronson N. (2013). Progression-Free Survival: What Does It Mean for Psychological Well-Being or Quality of Life? [Internet].

[36] Hwang T.J., Gyawali B. (2019). Association between progression-free survival and patients’ quality of life in cancer clinical trials. Int. J. Cancer.

[37] Plummer C., Michael A., Shaikh G., Stewart M., Buckley L., Miles T., Ograbek A., McCormack T. (2019). Expert recommendations on the management of hypertension in patients with ovarian and cervical cancer receiving bevacizumab in the UK. Br. J. Cancer.

[38] Dincer M., Altundag K. (2006). Angiotensin-converting enzyme inhibitors for bevacizumab-induced hypertension. Ann. Pharmacother..

[39] Economopoulou P., Kotsakis A., Kapiris I., Kentepozidis N. (2015). Cancer therapy and cardiovascular risk: Focus on bevacizumab. Cancer Manag. Res..

