# Supplementary figures and images for: Optimizing Outcomes: Bevacizumab with Carboplatin and Paclitaxel in 5110 Ovarian Cancer Patients—A Systematic Review and Meta-Analysis
Source: Pharmaceuticals (Basel). 2024 Aug 21;17(8):1095. doi: 10.3390/ph17081095 (PMC11359859; doi:10.3390/ph17081095)

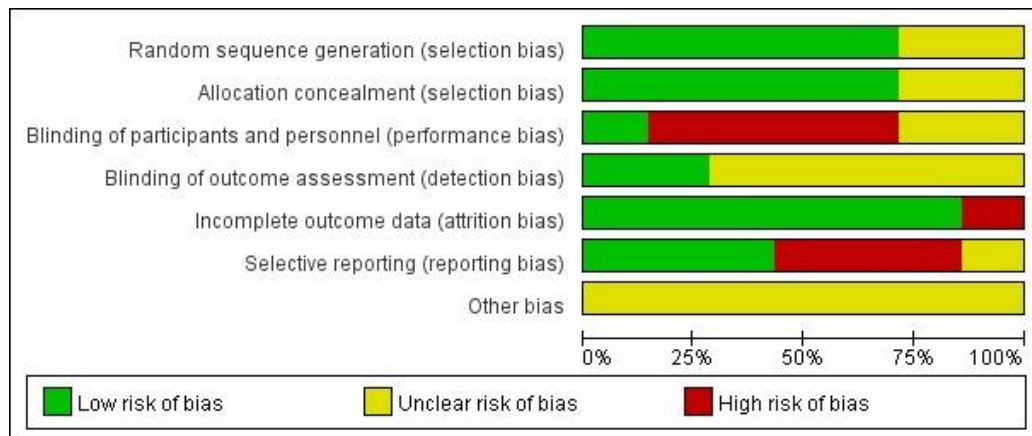

**Supplement Figure 1.** Risk of bias in Included studies.

Supplement: Supplementary file 1 [file pharmaceuticals-17-01095-s001.zip › pharmaceuticals-3101497-supplementary.pdf]
